# TAMA: improved metagenomic sequence classification through meta-analysis

**DOI:** 10.1186/s12859-020-3533-7

**Published:** 2020-05-12

**Authors:** Mikang Sim, Jongin Lee, Daehwan Lee, Daehong Kwon, Jaebum Kim

**Affiliations:** Department of Biomedical Science and Engineering, Konkuk University, Seoul, 05029 Republic of Korea

**Keywords:** Metagenome, Taxonomy analysis, Meta-analysis

## Abstract

**Background:**

Microorganisms are important occupants of many different environments. Identifying the composition of microbes and estimating their abundance promote understanding of interactions of microbes in environmental samples. To understand their environments more deeply, the composition of microorganisms in environmental samples has been studied using metagenomes, which are the collections of genomes of the microorganisms. Although many tools have been developed for taxonomy analysis based on different algorithms, variability of analysis outputs of existing tools from the same input metagenome datasets is the main obstacle for many researchers in this field.

**Results:**

Here, we present a novel meta-analysis tool for metagenome taxonomy analysis, called TAMA, by intelligently integrating outputs from three different taxonomy analysis tools. Using an integrated reference database, TAMA performs taxonomy assignment for input metagenome reads based on a meta-score by integrating scores of taxonomy assignment from different taxonomy classification tools. TAMA outperformed existing tools when evaluated using various benchmark datasets. It was also successfully applied to obtain relative species abundance profiles and difference in composition of microorganisms in two types of cheese metagenome and human gut metagenome.

**Conclusion:**

TAMA can be easily installed and used for metagenome read classification and the prediction of relative species abundance from multiple numbers and types of metagenome read samples. TAMA can be used to more accurately uncover the composition of microorganisms in metagenome samples collected from various environments, especially when the use of a single taxonomy analysis tool is unreliable. TAMA is an open source tool, and can be downloaded at https://github.com/jkimlab/TAMA.

## Background

Microbes are essential occupants in an ecological system that interact with and affect their environment. In the sea, these microbes help recycle nutrients [1]. In an alpine ecosystem, they compete for nitrogen with plants [2]. They also live in animal organs. They are thought to be the cause of many diseases [3–5]. They can affect the environment in various areas, such as animal development and biofuel production [6, 7]. Although it is crucial to identify the ecosystem of the microorganism in its environment, it is still hard to decipher the composition and functions of microbes in an environment because most bacteria on Earth cannot be cultivated [8].

Recently, the next-generation sequencing (NGS) technology has enabled studies of metagenomes [9], which are the sets of whole genetic materials of microorganisms in an environmental sample. Whole-genome sequence-level analysis of metagenomes is useful to research microbes in an environmental sample, including unculturable microbes. Taxonomy analysis using metagenomic reads has been used to identify the composition and abundance of the microorganisms in an environmental sample. Several methods have been developed for this purpose, including the k-mer-based approach and the read mapping-based approach. In k-mer-based taxonomy analysis tools, such as CLARK [10] and Kraken [11], all k-mers, which are possible substrings with length k in sequences, are extracted from both reference sequences and metagenome reads. Metagenome reads are then classified to reference sequences with the most similar k-mer composition. Read mapping-based taxonomy classification tools such as Centrifuge [12] assign metagenome reads to one or more taxons with the best mapping score against a compressed reference database.

Although there are many taxonomy classification tools, their results and performance are quite different, even with the same input metagenome read datasets [13, 14]. Therefore, it is difficult to know which taxonomy classification tool is the best for a given metagenome data [15]. In order to overcome such problem, meta-analysis approaches have recently been utilized to metagenome analysis [16–18]. The concept of meta-analysis was originally used in the clinical field for systematic and integrated studies of multiple findings from different sources [19–22]. In the metagenome analysis, the meta-analysis approach can be effectively used to remove both false positive and false negative analysis results, which can lead to the better understanding of the microbial community in environment. However, the application of the meta-analysis approaches to the metagenome analysis still lags behind.

Here, we present a novel taxonomy classification tool for metagenome NGS reads, called TAMA. TAMA performs the meta-analysis by integrating read assignment obtained from taxon ID classification with CLARK, Kraken, and Centrifuge using integrated reference database. A read classification profile is then generated by reassigning taxon ID(s) to each read based on the meta-analysis. Relative species abundance profile is next created using the read classification profile based on estimated genome size. TAMA outperformed existing taxonomy analysis tools in evaluation using simulated metagenome read datasets and the Critical Assessment of Metagenome Interpretation (CAMI) metagenome read datasets [23]. Relative species abundance profiles for real metagenome samples from two different cheese and human gut were then obtained, and differences in composition and abundance were identified using TAMA. TAMA will contribute to more accurately uncovering of the composition of microorganisms in metagenome samples collected from various environments, especially when the use of a single taxonomy analysis tool is unreliable. TAMA can be downloaded at https://github.com/jkimlab/TAMA together with an integrated reference database.

## Implementation

### Overview of TAMA

**Fig. 1:**
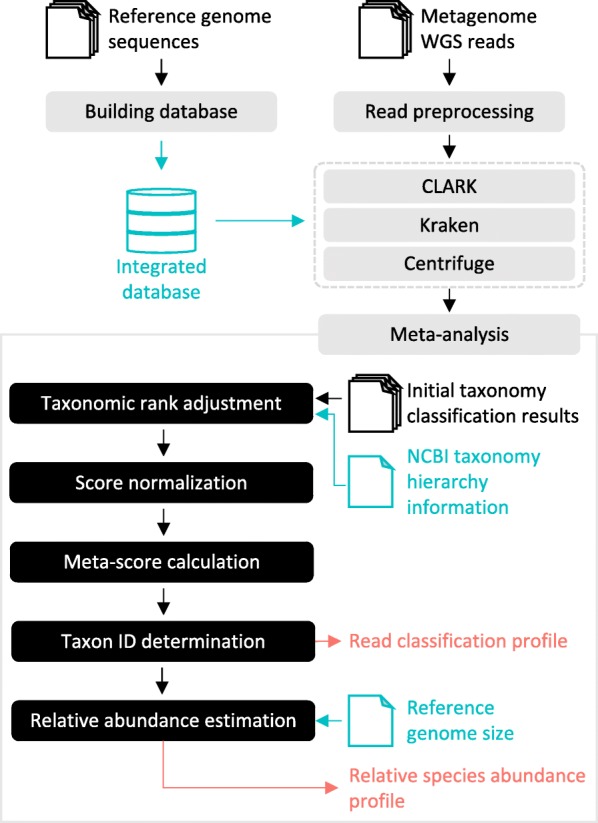
Overview of TAMA. In the read preprocessing step, low-quality input metagenome reads (single- or paired-end) are eliminated. Integrated database with identical set of reference genomes is also created. Initial taxonomy classification results, which has assigned taxon IDs for each read sequence, are generated by using CLARK, Kraken, and Centrifuge with the integrated database. In the meta-analysis process, results from the three tools are calibrated and integrated to produce a read classification and relative species abundance profile. The relative species abundance profile is generated only when the target taxonomic rank is species

TAMA is a meta-analysis tool for metagenome taxonomy analysis. Given multiple sets of metagenome reads, TAMA can perform taxonomy analysis by integrating analysis results from existing taxonomy analysis tools at user-specified target taxonomic rank (phylum, class, order, family, genus, or species). TAMA consists of three steps: read preprocessing, taxonomy analysis, and meta-analysis (Fig. 1). In the read preprocessing step, quality control of read sequences is performed. In the taxonomy analysis step, taxon IDs are assigned to reads using three taxonomy analysis tools, CLARK [10], Kraken [11], and Centrifuge [12], based on an integrated database of bacterial genome sequences. These three tools were selected because their performance was good based on literature survey, and it was easy to use them and easy to customize their database. In the meta-analysis step, results from the three tools are calibrated and integrated to reassign a final taxon ID for each read, and a relative species abundance profile is produced. The details of each step are described in the following subsections.

### Read preprocessing step

Trimmomatic (v0.36) [24] and BayesHammer [25] are used in this step to perform metagenome read quality control. Trimmomatic removes or trims low-quality reads, while BayesHammer corrects sequencing errors. TAMA supports both single- and paired-end reads.

### Taxonomy analysis step

Three taxonomy analysis tools (CLARK, Kraken, and Centrifuge) are used in each high-quality metagenome read to assign a single or multiple taxon ID(s) (per read in the case of a single-end read, and per read pair in the case of a paired-end read). Because each of the three tools needs to use a different type of database of bacterial genome sequences, a database is generated for each taxonomy analysis tool separately (collectively called the TAMA database) using the same set of bacterial reference genome sequences (total 111,029) downloaded from the NCBI RefSeq website (https://www.ncbi.nlm.nih.gov/refseq/) in Nov. 2017. Supplementary Table S1 shows detailed information of reference sequences. The above three taxonomy analysis tools were executed with their default parameter values in this study.

### Meta-analysis step

The meta-analysis step integrates and reassigns all classified results from the three taxonomy analysis tools, followed by the creation of a read classification profile and a relative species abundance profile. The meta-analysis step has five sub-steps: taxonomic rank adjustment, score normalization, meta-score calculation, taxon ID determination, and relative species abundance estimation.

First, in the taxonomic rank adjustment step, initially assigned taxon IDs of a read from each taxonomy analysis tool are adjusted up to a user-provided target taxonomic rank based on taxonomic hierarchy information obtained from the NCBI taxonomy database (https://www.ncbi.nlm.nih.gov/taxonomy). Because Kraken and Centrifuge automatically assign the best possible taxon IDs out of the six taxonomic ranks to a read instead of following a given target taxonomic rank, this step is applied only to the results of Kraken and Centrifuge. In the case of CLARK, this step is not used because it takes the target taxonomic rank as input, and generates results based on the target taxonomic rank. This taxonomic rank adjustment is possible only when the taxonomic rank of the initially assigned taxon ID is lower than the target taxonomic rank. Otherwise, because a specific taxon ID at a lower taxonomic rank cannot be determined, the initially assigned taxon ID is ignored and the corresponding read is labeled as unclassified. If a read does not have any assigned taxon ID in the previous taxonomy analysis step, it is also labeled as unclassified.

Because the scale of assignment confidence scores from these three tools is different, all taxon assignment scores for adjusted taxon IDs in a read are normalized with eq. (1) in the score normalization step:

1 $$ {S}_{r,i,t}={s}_{r,i.t}\ast \frac{N_{r,i,t}}{N_{r,i}} $$

where *S*_*r*, *i*, *t*_ is the normalized taxon assignment score of an adjusted taxon *t* to a read *r* from the taxonomy analysis tool i ∈ {CLARK, Kraken, Centrifuge}, *s*_*r*, *i*. *t*_ is a min-max normalized score over the range [0,1] of the original assignment score of the adjusted taxon *t* obtained from the tool *i*, *N*_*r*, *i*_ is the total number of assigned taxon IDs to the read *r* obtained from the tool *i*, and *N*_*r*, *i*, *t*_ is the number of adjusted taxon ID *t* assigned to the read *r* obtained from the tool *i*. CLARK and Kraken always produce a single taxon ID for one read. Therefore, *N*_*r*, *i*, *t*_ and *N*_*r*, *i*_ are always 1. However, if their assignment scores are equally the best, Centrifuge can assign multiple taxon IDs to a single read. Therefore, originally assigned different taxon IDs can be adjusted to the same taxon at higher taxonomic rank, leading to multiple existence of the same taxon ID assigned to a single read (*N*_*r*, *i*, *t*_ > 1). In this case, the last term *N*_*r*, *i*, *t*_/*N*_*r*, *i*_ in equation (1) contributes to distributing *s*_*r*, *i*. *t*_ to each differently adjusted taxon ID proportional to its fraction against *N*_*r*, *i*_. This strategy is used to prevent overestimation of an assigned taxon resulting from (i) duplicate assignment of the same taxon to a single read or (ii) the nature of the final meta-score, which is the sum of taxon assignment scores from the three tools that will be described in the following subsection. For all “unclassified” reads in the previous taxonomy analysis step, *S*_*r*, *i*, *t*_ is set to 0.

Normalized scores (*S*_*r*, *i*, *t*_) from the three tools are integrated to calculate the meta-score *M*_*r*, *t*_ shown in equation (2) in the meta-score calculation step:

2 $$ {M}_{r,t}=\frac{1}{F}\sum \limits_i\left({S}_{r,i,t}\ast {F}_i\right) $$

where *F*_*i*_ is the weight of a tool *i* representing the relative performance of the three tools, and *F* is the sum of all *F*_*i*_ s. Default values of *F*_*i*_ are set to 1 for all tools, but user can change those values. In the taxon ID determination step, taxon IDs with the highest *M*_*r*, *t*_ are reassigned, resulting in the generation of the read classification profile for each read.

Finally, when the target taxonomic rank is species, a relative species abundance profile is estimated by using the read classification profile. Before calculating the relative species abundance, additional filtering is performed by using the meta-score. For all assigned species, the average meta-score is computed and the species with an average meta-score less than 0.34 is ignored in abundance estimation. The default cutoff score of 0.34 was empirically estimated using simulated metagenome datasets, and can be changed by user. The estimated relative species abundance *EA*_*s*_ is calculated with equation (3):

3 $$ {EA}_s=\left({RC}_s/{GS}_s\right)/\sum \limits_i\left({RC}_i/{GS}_i\right) $$

where RC_*s*_ and GS_*s*_ represent the number of assigned reads to a species *s* and the average genome size of the species, respectively; while ${\sum}_i\left({\mathrm{RC}}_i/{\mathrm{GS}}_i\right)$ represents the sum of the number of reads over the genome size of all species with classified reads. Therefore, *EA*_*s*_ represents only the relative proportion among all species with classified reads. The average genome size of each species is calculated using all sub-strain and sub-species genomes in known reference genome sequences. This is because there can be many genome sequences with different genome sizes for the same species. For each reference genome, its genome size was calculated using both chromosome and plasmid sequences.

### Simulated metagenome read generation for performance evaluation

Metagenome read datasets were simulated to have two types of reads: (i) generated from bacterial genome sequences which were treated as true positive reads, and (ii) random reads simulated by using fabricated sequences from non-bacterial genome sequences which were treated as true negative reads. Specifically, metagenome profiles were first generated using different numbers of genomes (10, 50, and 100) to simulate bacterial read sequences of metagenome read datasets. For each dataset with a total of *N* genomes, Poisson distribution was utilized with different parameter values to create a diverse abundance of *N* genomes. For 10 genomes dataset, six different parameter values (0.1, 0.5, 1.25, 2.5, 3.75, and 5) were used to generate the abundance profile. For 50 and 100 genomes datasets, seven different parameter values of (0.1, 0.5, 2.5, 6.25, 12.5, 18.75, and 25) and (0.1, 0.5, 5, 12.5, 25, 37.5, and 50) were used, respectively (Supplementary Tables S2–S4). By randomly assigning species selected from the NCBI RefSeq bacterial genome sequences (a total of 2788 genomes downloaded in Dec. 2016) without replacement to those 20 abundance profiles five times, 100 different profiles of species abundance were generated.

Once species abundance profiles were created, read sequences were generated using the read simulation program ART [26] based on the Illumina HiSeq 2500 sequencing platform with 101 bp read length and 500 bp insert size. In this read simulation, the number of simulated reads of each chosen species was calculated considering its assigned abundance and genome size as defined in equation (4):

4 $$ {R}_s=\frac{\left({A}_s\ast {G}_s\right)\ast T}{\sum \limits_k\left({A}_k\ast {G}_k\right)} $$

where *R*_*s*_, *A*_*s*_, *G*_*s*_, and *T* are the number of reads, the abundance, the genome size of species *s*, and the total number of reads (6,000,000 in this study), respectively.

Random reads were downloaded from a recent benchmarking study for metagenome analysis tools [15]. From whole random read sequences, 100 different sets of 600,000 read sequences were randomly extracted and added into the simulated metagenome read datasets.

### Evaluation of read classification performance

For each of the simulated metagenome read dataset, the assignment accuracy was measured using the recall, precision, and F1-score measures. To calculate recall and precision scores, numbers of true positive (TP), true negative (TN), false positive (FP), and false negative (FN) were counted from a read dataset as follows. When the taxon ID was correctly assigned for the simulated read, then the taxon ID was counted as TP, otherwise as FN. Also, when the taxon ID was precisely unassigned for the random read, then the taxon ID was counted as TN, otherwise as FP. In the case of Centrifuge, multiple taxon IDs can be assigned to a single read. Therefore, if a simulated read was assigned to several different taxon IDs and only some of them were right, proportions of right and wrong assignment were used as TP and FN, respectively. These four numbers were then used to calculate recall (TP/(TP + FN)) and precision (TP/(TP + FP)) scores, which were then used to calculate the F1-score with equation (5):

5 $$ \mathrm{F}1-\mathrm{score}=2\ast \frac{\mathrm{recall}\ast \mathrm{precision}}{\mathrm{recall}+\mathrm{precision}} $$

### Evaluation of species prediction and abundance estimation

To assess the performance of species prediction, we compared the list of species in between the read simulation profile and the estimated relative species abundance profile for each simulated dataset. We measured the performance with recall, precision, and F1-score. In the predicted abundance profile from Centrifuge, some species were included in the profile with zero abundance (or ratio). We considered them as nonexistent species and ignored them for the comparison.

To evaluate the estimated relative species abundance, we calculated the sum of relative abundance difference compared to the true relative abundance profile. The sum of the relative abundance difference was calculated with equation (6):

6 $$ \mathrm{SDra}=\sum {O}_x+\sum {T}_y+\sum {P}_z. $$

where *O*_*x*_, *T*_*y*_, and *P*_*z*_ are relative abundance difference of species *x* which exists in both true relative abundance profile and the predicted abundance profile, the relative abundance of species *y* is only observed in the true relative abundance profile, and the relative abundance of species *z* is only present in the predicted abundance profile, respectively. When the set of predicted species and their relative abundance completely agree with the true answer, the SDra score becomes the minimum (the best) score of 0. When there is no overlap between species in the predicted and true relative abundance profile, it becomes the maximum (the worst) score of 2.

### Real metagenome read datasets

Real metagenome read datasets were downloaded from the EBI metagenome database (https://www.ebi.ac.uk/metagenomics). Two cheese metagenomes (artisanal cheese: ERP004234, cotija cheese: SRP059999) and one human gut metagenome (ERP002469) were collected (Supplementary Table S5). Their compositions and relative species abundance were identified using TAMA.

## Results

### Evaluation of TAMA using simulated metagenome datasets

The performance of TAMA was evaluated by comparing to the three initial taxonomy analysis tools (CLARK [10], Kraken [11], and Centrifuge [12]) used in TAMA in terms of the accuracy of read classification, species prediction, and species abundance estimation using simulated datasets (Implementation).

**Fig. 2:**
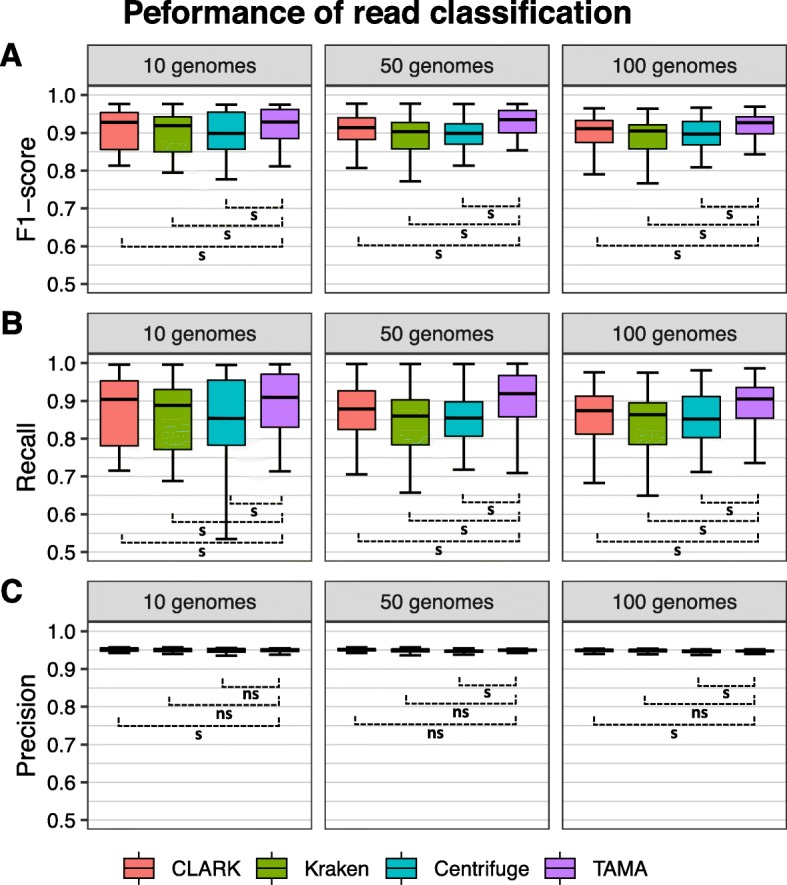
Performance evaluation results of read classification for the species rank. Boxplots indicate the distribution of (**a**) F1-score, (**b**) recall, and (**c**) precision of read classification in the 10 genomes dataset (left), 50 genomes dataset (center), and 100 genomes dataset (right). The Wilcoxon signed-rank test was used for pairwise comparison between TAMA and the others (s: *p*-value <= 0.05, and ns: *p*-value > 0.05)

Firstly, the performance of read classification was measured by calculating F1-score at species rank (Fig. 2a). For all three types of genome datasets, TAMA showed the best performance (the highest classification accuracy with the smallest variance) compared to the initial three taxonomy analysis tools. In the examination of recall and precision (Fig. 2b, c), TAMA successfully increased recall while preserving precision. These evaluations were also repeated for other taxonomic ranks, and similar performance of TAMA was observed (Supplementary Figs. S1-S5).

**Fig. 3:**
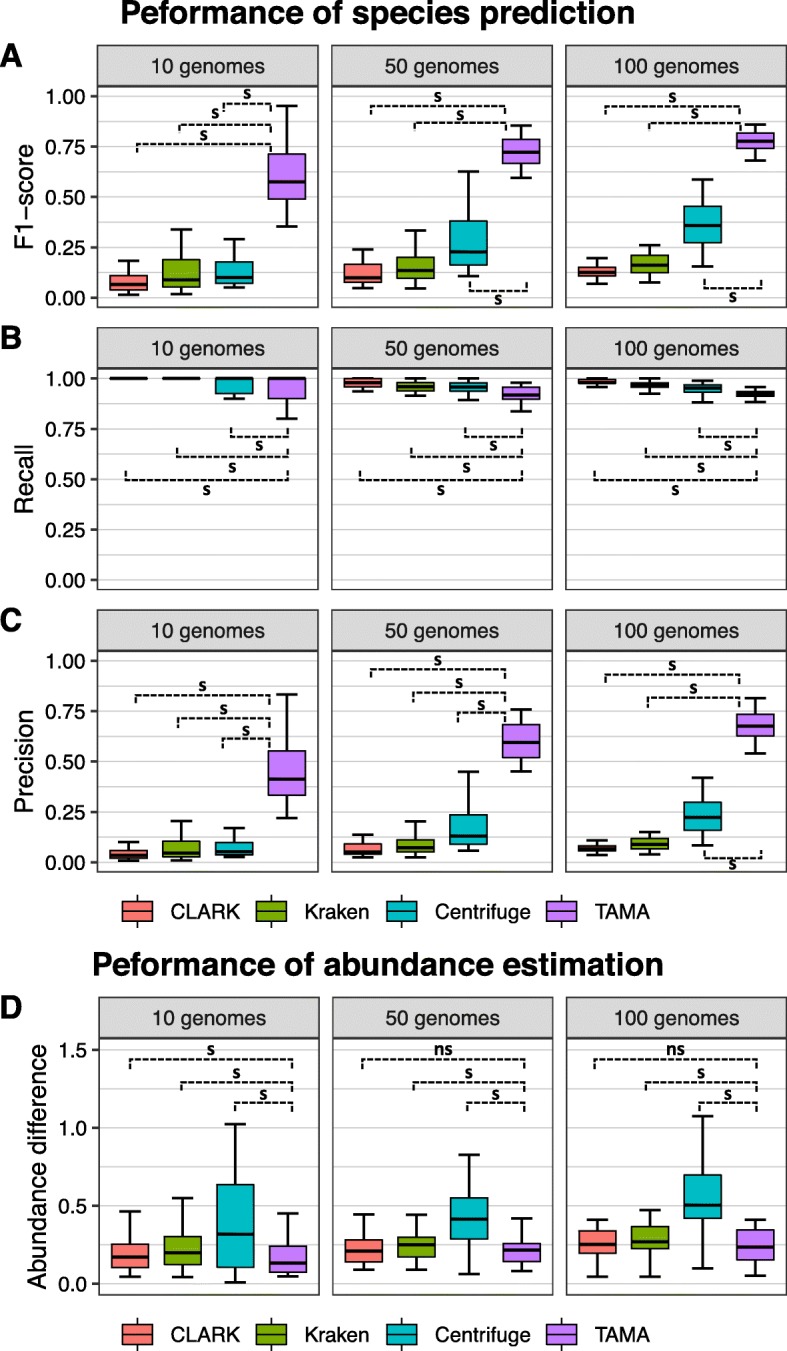
Performances of initial taxonomy analysis tools and TAMA in species identification and abundance estimation. Boxplots indicate the distribution of (**a**) F1-score, (**b**) recall, and (**c**) precision of species prediction and (**d**) the sum of abundance differences of all species in the 10 genomes dataset (left), 50 genomes dataset (center), and 100 genomes dataset (right). The Wilcoxon signed-rank test was used for pairwise comparison between TAMA and the others (s: *p*-value <= 0.05, and ns: *p*-value > 0.05)

To assess the performance of TAMA in terms of species prediction, we compared simulated and predicted abundance profiles by the four tools, including TAMA, in terms of the F1-score, recall, and precision (Fig. 3a-c). TAMA successfully improved the performance of species prediction for all types of datasets. Specifically, TAMA dramatically increased the precision with minor decrease of recall in all types of datasets. The recall of all tools was less variable and high (≥ 0.8), whereas their precision was highly variable (Fig. 3b, c).

Finally, the identified species by each tool were more deeply examined and compared in terms of relative species abundance. The abundance difference of each species was first calculated by comparing the predicted relative abundance of the species by each tool with the true answer in the simulated datasets. Abundance differences from all species were then added (see Implementation). Note that the relative species abundance of CLARK or Kraken was generated using the abundance estimation module of TAMA because neither CLARK nor Kraken could directly generate relative species abundance (they could only generate the proportion of reads for each assigned species). For all datasets, the overall performance of TAMA was superior to all three tools in all datasets (Fig. 3d). More obvious performance gap was observed when the numbers of samples with the smallest abundance difference were compared (Table 1 and Supplementary Table S6). For example, in the 50 genomes dataset, TAMA showed the smallest abundance difference in 21 out of total 35 samples, whereas the abundance was the smallest only in 8, 1, and 5 samples in the case of CLARK, Kraken, and Centrifuge respectively.

**Table 1:** The number of samples with the minimum abundance difference

Dataset	10 genomes	50 genomes	100 genomes
No. of samples	30	35	35
CLARK	2	8	11
Kraken	2	1	1
Centrifuge	9	5	3
TAMA	17	21	20

### Evaluation of TAMA compared to another meta-analysis tool

**Fig. 4:**
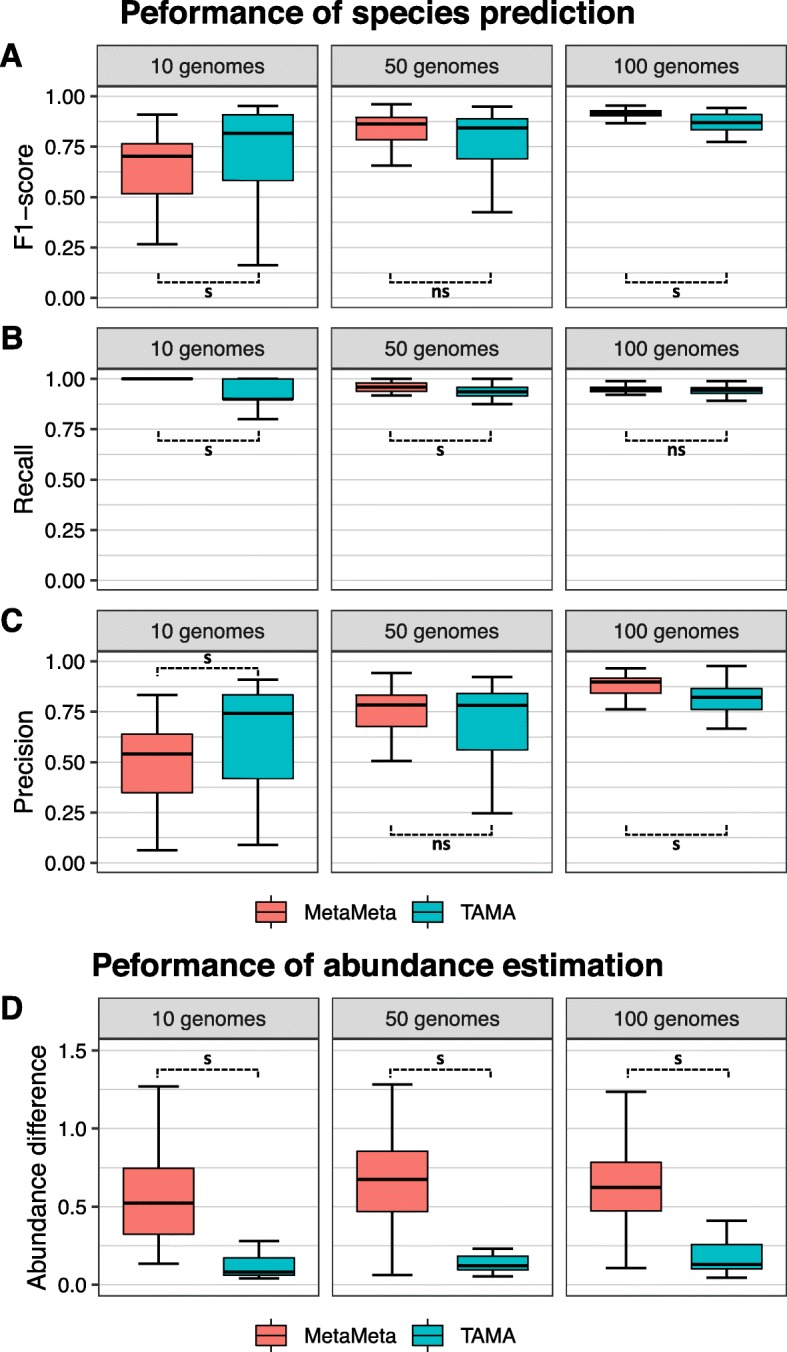
Performance of meta-analysis tools in species identification and abundance estimation. Boxplots indicate the distribution of (**a**) F1-score, (**b**) recall, and (**c**) precision of species prediction and (**d**) the sum of abundance differences of all species in the 10 genomes dataset (left), 50 genomes dataset (center), and 100 genomes dataset (right). The Wilcoxon signed-rank test was used for pairwise comparison between TAMA and MetaMeta (s: *p*-value <= 0.05, and ns: *p*-value > 0.05)

We evaluated the performance of TAMA compared to another meta-analysis tool, MetaMeta [16]. When we compared TAMA with MetaMeta, we used the original reference database of MetaMeta for comparison of MetaMeta and TAMA. Specifically, we used the Kraken database in MetaMeta as itself and created CLARK reference database using the list of reference genomes of CLARK from the MetaMeta database information. Taxonomy analysis tools that are overlapped with MetaMeta and TAMA are only CLARK and Kraken. Thus, we also created Centrifuge reference database for the integrated reference database by using the list of reference in CLARK. We used identical measures to previous evaluation of species prediction and abundance estimation using simulated metagenome datasets. TAMA performed better than MetaMeta in the case of the 10 genomes dataset, but showed slightly worse performance in the case of the 100 genomes dataset (Fig. 4). However, TAMA achieved dramatically smaller abundance difference than MetaMeta for all the three datasets.

### Evaluation of TAMA using CAMI metagenome datasets

Critical Assessment of Metagenome Interpretation (CAMI) data [23] was used for separate evaluation at species rank. In this evaluation, one sample from each of low (only one sample exists), medium (the sample2 from total two samples), and high (the sample3 from total five samples) complexity dataset in CAMI was used to compare the performance for species identification and their abundance estimation of CLARK, Kraken, Centrifuge, and TAMA. The CAMI dataset provides the list of genomes, their corresponding taxon IDs, and their relative abundance that were used to simulate the datasets. However, there is no information of a true taxon ID for each read. Therefore, we only compared the performance in terms of species prediction and abundance estimation. As in the previous section, relative species abundances of CLARK and Kraken were created by using the abundance estimation module of TAMA.

Similar patterns to the previous evaluation were observed (Table 2). In the performance of species prediction, TAMA showed similar recall values compared to other tools. However, it showed extremely higher precision values, leading to a dramatic increase of F1-score compared to other tools. Precision was increased more than ten times by using TAMA compared to the use of non-meta-analysis tools for all complexity datasets. We also obtained more than four times higher F1-score in species prediction with TAMA compared to the best of other tools. The performance of abundance difference of all tools was very similar.

**Table 2:** Performance evaluation results using CAMI metagenome datasets

Sample		Species prediction			Abundance difference
		Precision	Recall	F1-score	
Low	CLARK	0.003	0.571	0.007	1.591
	Kraken	0.003	0.524	0.007	1.620
	Centrifuge	0.044	0.524	0.082	1.627
	TAMA	0.917	0.524	0.667	1.620
Medium	CLARK	0.010	0.486	0.019	0.800
	Kraken	0.010	0.486	0.019	0.800
	Centrifuge	0.058	0.486	0.104	0.893
	TAMA	0.652	0.429	0.517	0.826
High	CLARK	0.022	0.336	0.042	1.198
	Kraken	0.022	0.336	0.042	1.198
	Centrifuge	0.071	0.328	0.117	1.256
	TAMA	0.720	0.320	0.443	1.207

### Application to cheese metagenomes

TAMA was applied to predict species and their relative abundance in two different cheese metagenomes (Artisanal and Cotija cheese). Highly variable relative abundance of different species was observed in different metagenome samples (Table 3 and Supplementary Tables S7, S8). The number and type of validated species between two cheese metagenome samples were very different. For example, the numbers of identified species for Artisanal cheese and Cotija cheese were 33 and 60, respectively. Moreover, among all predicted profiles, only eight species (*Leuconostoc mesenteroides*, *Aerococcus viridans*, *Enterococcus faecium*, *Enterococcus italicus*, *Enterococcus faecalis*, *Lactococcus lactis*, *Streptococcus macedonicus*, and *Streptococcus thermophilus*) were overlapped in both cheese metagenomes. To identify the distribution of relative abundance, we counted the number of species occupied more than 50% (P50) and more than 90% (P90). We found that both cheese metagenome had a few dominant species that occupied a large proportion of each sample. In the case of Artisanal cheese, *Mycobacterium malmesburyense* and *Streptococcus macedonicus* showed large relative abundances (more than 0.66 and 0.26 of the sample, respectively).

**Table 3:** Summary of metagenome profiles

	Artisanal cheese	Cotija cheese	Human gut
No. of species	34	60	80
P50^a^	1	3	7
P90^b^	2	10	26

^a^The number of species occupied more than 50% of relative abundance

^b^The number of species occupied more than 90% of relative abundance

### Application to human gut metagenome

**Fig. 5:**
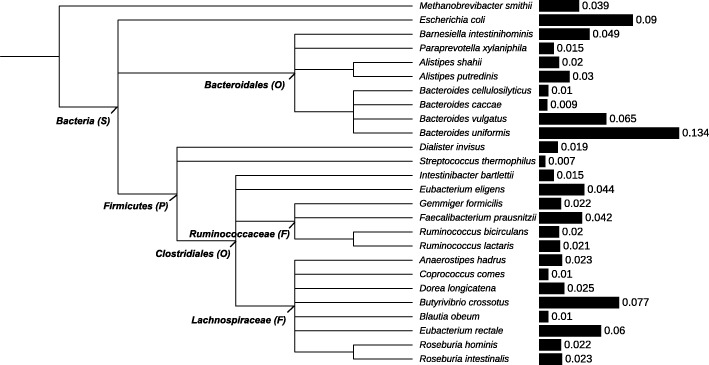
Phylogenetic tree of the identified human gut metagenome. It indicates 26 species included in P90 of identified species at leaf nodes and their upper groups (S: superkingdom, P: phylum, O: order, and F: family). The relative species abundance is represented the bar plot with the value shown at the right side

We identified a metagenome profile of human gut sample using TAMA. In the human gut metagenome sample, 3,380,409 reads were classified to species in the reference database, 80 species were identified, and 7 species occupied more than 50% (Table 3 and Supplementary Table S9). There was no species that had an occupancy of more than 15% in the sample. The most abundant species was *Bacteroides uniformis* (0.13), followed by *Escherichia coli* (0.09). We constructed a phylogenetic tree with 26 species that were included in P90 and indicated their relative abundances using iTOL [27]. *Bacteroidales* and *Clostridiales* occupied approximately 33% and 41% of predicted species, respectively. Approximately 9 % of identified species were *Escherichia coli* in the human gut metagenome sample (Fig. 5).

## Discussion

TAMA is a novel meta-analysis tool for metagenome taxonomy analysis. TAMA provides classification results from the output of different taxonomy analysis tools by integrating taxon ID assignment of reads and generating an improved relative species abundance profile. Taxonomy classification can be performed for multiple types of sequence files from a sample and for multiple numbers of metagenome samples at once. To reduce any bias from reference databases in the classification for each taxonomy analysis tool, an integrated reference database was generated and embedded in each tool. We provide an integrated reference database with the NCBI RefSeq genome sequences. In addition, users can easily create the integrated database using their reference genome sequences. Taxonomy classification result can be obtained for one of six taxonomy ranks (phylum, class, order, family, genus, and species). A relative species abundance profile can be generated using the estimated genome size of each species for the species rank classification.

To evaluate TAMA, various types of simulated metagenome read datasets were generated. They were composed of various numbers of genomes and distributions of their relative abundance to cover as many different conditions of metagenome samples as possible. TAMA was compared to three non-meta-analysis tools (CLARK, Kraken, and Centrifuge) and one meta-analysis tool (MetaMeta). MetaMeta is based on six different taxonomy analysis tools (CLARK [10], DUDes [28], GOTTCHA [29], Kaiju [30], Kraken [11], mOTUs [31]), while two of them (CLARK and Kraken) overlap with TAMA. The reference database could not be fully customized for every tool used in MetaMeta. Therefore, a similar reference database to the one used in MetaMeta was created and used for fair evaluation.

The main difference between MetaMeta and TAMA is in the way of integrating results from different tools. TAMA uses an eager integration scheme in the sense that the read classification results from used tools are integrated which is followed by the prediction of species and their abundance. However, MetaMeta relies on a lazy integration scheme because it allows used tools to classify reads and predict species abundance separately, and then integrates the final results. In addition, whereas MetaMeta filters out only species with low abundance in the final integrated results, TAMA can remove reads with low confidence score before predicting species and their abundance. The eager integration scheme with the use of classified reads with high confidence may lead to higher performance of TAMA than MetaMeta especially in the estimation of species abundance, indicating that a result integration scheme may be a more important factor than the number of used tools in the meta-analysis.

In the evaluation with simulated metagenome datasets, we first examined each assignment of read. As shown in Fig. 2, all initial taxonomy analysis tools and TAMA showed F1-score of more than 0.75. However, TAMA had the smallest variance and the highest median for all datasets. We performed the Wilcoxon signed-rank test to examine whether the performance of TAMA was significantly greater than performances of initial tools. From the analysis, we obtained significantly low *p*-value (< 0.05 for all pairs by the Wilcoxon signed-rank test), indicating that statistically significant performance improvement was possible with TAMA. Additionally, because we provide all assigned taxon IDs in read classification profile with high accuracy and the score of meta-analysis, we believe that TAMA could be helpful in a following study utilizing classified read information.

The performance of TAMA was then examined in terms of the accuracy of predicting existing species in a metagenome dataset. We reduced many false positive species from the predicted species with meta-score of TAMA, indicating a degree of confidence for an integration. In the evaluation using the CAMI metagenome, non-meta-analysis tools had very low predictive precisions of species. This has been similarly observed in a previous study at species rank [32]. However, TAMA was able to increase the precision more than ten times. A tiny amount of incorrectly assigned reads are one of the reasons for the generation of false positively predicted species. Another reason is that a part of the read sequence for a single genome could be generated from the common sequence region between different genomes. We could filter out assignments that have low confidence using the meta-score of TAMA by calculating the average meta-score of the predicted species and ignoring the unreliable prediction with the average. It is considered to be an advantage of meta-analysis because MetaMeta, another meta-analysis program, also shows similar performance in species prediction. Moreover, TAMA shows improved performance in the estimation of relative species abundance, in line with its read classification performance.

Finally, TAMA was applied to predict species and their abundances in real metagenome samples from various environments. In the investigation of three real metagenome samples, it was found that compositions and relative abundances of species were very different in different environments. In Artisanal cheese, there are dominantly existing species (the relative abundances of a species > 0.5). However, other metagenome samples do not have such dominant species. TAMA could successfully identify three main bacterial genomes in the Cotija cheese sample, namely *Lactobacillus plantarum*, *Weissella paramesenteroides*, and *Leuconostoc mesenteroides* [33]. There are some limitations to apply it to real metagenome datasets. There are still many unclassified read sequences because of the shallow coverage of the reference database (Supplementary Tables S7-S9). This is a common problem in taxonomy analysis based on a reference database. This will be alleviated as more and more new species are discovered and added into the reference database.

One drawback of TAMA is relatively long runtime because of the requirement of running multiple taxonomy analysis tools. However, we believe that it can be complemented by modern computing power, such as the capability of parallel computing, and superior performance of TAMA compared to other tools. As a future direction, TAMA will become more customizable to use any number of taxonomy analysis tools chosen by users, and the integrated reference sequence database in TAMA will be kept updated to reflect the changes of bacterial genome sequences in the NCBI database.

## Conclusion

TAMA is a meta-analysis tool for the taxonomy analysis of metagenome reads at the user selected taxonomic rank. TAMA can be used to improve the quality of taxonomy classification profiles, and to reduce many of the false positives. We believe that TAMA is the most accurate and easy-to-use existing meta-analysis tool based on evaluation results in comparison with other tools, and TAMA can contribute to more accurate metagenome analysis if more accurate and larger amounts of reference genomes are accumulated.

## Availability and requirements

Project name: TAMA.

Project home page: https://github.com/jkimlab/TAMA

Operating system: Linux.

Programming language: Perl.

Other requirements: Docker.

License: MIT.

Any restrictions to use by non-academics: License needed.

## Supplementary information

**Additional file 1: Supplementary Table S1.** The statistics of reference sequences used in the TAMA database. **Supplementary Table S2.** The relative abundance of 10 genomes generated using six different parameter values of the Poisson distribution. **Supplementary Table S3.** The relative abundance of 50 genomes generated using seven different parameter values of the Poisson distribution. **Supplementary Table S4.** The relative abundance of 100 genomes generated using seven different parameter values of the Poisson distribution. **Supplementary Table S5.** The statistics of read sequences in real metagenome datasets. **Supplementary Table S6.** The abundance difference of initial taxonomy analysis tools and TAMA for the simulated metagenome datasets. **Supplementary Table S7.** The abundance profile of Artisanal cheese metagenome. **Supplementary Table S8.** The abundance profile of Cotija cheese metagenome. **Supplementary Table S9.** The abundance profile of human gut metagenome.

**Additional file 2: Supplementary Figure S1.** Performance evaluation results of read classification for the genus rank. Boxplots indicate the distribution of (A) F1-score, (B) recall and (C) precision of read classification in 10 genomes dataset (left), 50 genomes dataset (center), and 100 genomes dataset (right). **Supplementary Figure S2.** Performance evaluation results of read classification for the family rank. Boxplots indicate the distribution of (A) F1-score, (B) recall and (C) precision of read classification in 10 genomes dataset (left), 50 genomes dataset (center), and 100 genomes dataset (right). **Supplementary Figure S3.** Performance evaluation results of read classification for the order rank. Boxplots indicate the distribution of (A) F1-score, (B) recall and (C) precision of read classification in 10 genomes dataset (left), 50 genomes dataset (center), and 100 genomes dataset (right). **Supplementary Figure S4.** Performance evaluation results of read classification for the class rank. Boxplots indicate the distribution of (A) F1-score, (B) recall and (C) precision of read classification in 10 genomes dataset (left), 50 genomes dataset (center), and 100 genomes dataset (right). **Supplementary Figure S5.** Performance evaluation results of read classification for the phylum rank. Boxplots indicate the distribution of (A) F1-score, (B) recall and (C) precision of read classification in 10 genomes dataset (left), 50 genomes dataset (center), and 100 genomes dataset (right).

## Data Availability

The package presented in this manuscript is available at: https://github.com/jkimlab/TAMA. The integrated databases and simulated metagenome datasets are available at: http://bioinfo.konkuk.ac.kr/TAMA. The CAMI datasets are available at: https://data.cami-challenge.org. The real metagenome datasets are available at: https://www.ebi.ac.uk/metagenomics. Project IDs are available at the Supplementary Table S5.

## Abbreviations

CAMI: Critical Assessment of Metagenomic Interpretation
TAMA: Taxonomy Analysis pipeline for metagenome using Meta-Analysis
