# Supplementary material for: TAMA: improved metagenomic sequence classification through meta-analysis
Source: BMC Bioinformatics. 2020 May 12;21:185. doi: 10.1186/s12859-020-3533-7 (PMC7218625; doi:10.1186/s12859-020-3533-7)
Supplement: Supplementary file 2 — Additional file 2: Supplementary Figure S1. Performance evaluation results of read classification for the genus rank. Boxplots indicate the distribution of (A) F1-score, (B) recall and (C) precision of read classification in 10 genomes dataset (left), 50 genomes dataset (center), and 100 genomes dataset (right). Supplementary Figure S2. Performance evaluation results of read classification for the family rank. Boxplots indicate the distribution of (A) F1-score, (B) recall and (C) precision of read classification in 10 genomes dataset (left), 50 genomes dataset (center), and 100 genomes dataset (right). Supplementary Figure S3. Performance evaluation results of read classification for the order rank. Boxplots indicate the distribution of (A) F1-score, (B) recall and (C) precision of read classification in 10 genomes dataset (left), 50 genomes dataset (center), and 100 genomes dataset (right). Supplementary Figure S4. Performance evaluation results of read classification for the class rank. Boxplots indicate the distribution of (A) F1-score, (B) recall and (C) precision of read classification in 10 genomes dataset (left), 50 genomes dataset (center), and 100 genomes dataset (right). Supplementary Figure S5. Performance evaluation results of read classification for the phylum rank. Boxplots indicate the distribution of (A) F1-score, (B) recall and (C) precision of read classification in 10 genomes dataset (left), 50 genomes dataset (center), and 100 genomes dataset (right). [file 12859_2020_3533_MOESM2_ESM.docx]

**Supplementary Figures**

Supplementary Figure S1. Performance evaluation results of read classification for the genus rank. Boxplots indicate the distribution of (A) F1-score, (B) recall and (C) precision of read classification in 10 genomes dataset (left), 50 genomes dataset (center), and 100 genomes dataset (right).

Supplementary Figure S2. Performance evaluation results of read classification for the family rank. Boxplots indicate the distribution of (A) F1-score, (B) recall and (C) precision of read classification in 10 genomes dataset (left), 50 genomes dataset (center), and 100 genomes dataset (right).

Supplementary Figure S3. Performance evaluation results of read classification for the order rank. Boxplots indicate the distribution of (A) F1-score, (B) recall and (C) precision of read classification in 10 genomes dataset (left), 50 genomes dataset (center), and 100 genomes dataset (right).

Supplementary Figure S4. Performance evaluation results of read classification for the class rank. Boxplots indicate the distribution of (A) F1-score, (B) recall and (C) precision of read classification in 10 genomes dataset (left), 50 genomes dataset (center), and 100 genomes dataset (right).

Supplementary Figure S5. Performance evaluation results of read classification for the phylum rank. Boxplots indicate the distribution of (A) F1-score, (B) recall and (C) precision of read classification in 10 genomes dataset (left), 50 genomes dataset (center), and 100 genomes dataset (right).
